# Tepid sponging plus dipyrone versus dipyrone alone for reducing body temperature in febrile children

**DOI:** 10.1590/S1516-31802008000200008

**Published:** 2008-03-06

**Authors:** João Guilherme Bezerra Alves, Natália Dornelas Câmara Marques de Almeida, Camila Dornelas Câmara Marques de Almeida

**Keywords:** Fever, Dipyrone, Child, Baths, Antipyretics, Febre, Dipirona, Criança, Banhos, Antipiréticos

## Abstract

**CONTEXT AND OBJECTIVE:**

The role of tepid sponging to promote fever control in children is controversial. We did not find any studies reporting on the effectiveness of tepid sponging in addition to dipyrone. The aim of this study was to compare the effects of tepid sponging plus dipyrone with dipyrone alone for reducing fever.

**DESIGN AND SETTING:**

A randomized clinical trial was undertaken at Instituto Materno-Infantil Professor Fernando Figueira, Recife, Pernambuco.

**METHODS:**

Children from six months to five years old with axillary temperature greater than 38 °C in the emergency ward between January and July 2006 were eligible. One hundred and twenty children were randomly assigned to receive oral dipyrone (20 mg/kg) or oral dipyrone and tepid sponging for 15 minutes. The primary outcome was mean temperature reduction after 15, 30, 60, 90 and 120 minutes. Secondary outcomes were crying and irritability.

**RESULTS:**

106 children finished the study. After the first 15 minutes, the fall in axillary temperature was significantly greater in the sponged group than in the control group (p < 0.001). From 30 to 120 minutes, better fever control was observed in the control group. Crying and irritability were observed respectively in 52% and 36% of the sponged children and in none and only two of the controls.

**CONCLUSIONS:**

Tepid sponging plus dipyrone cooled faster during the first 15 minutes, but dipyrone alone presented better fever control over the two-hour period. Tepid sponging caused mild discomfort, crying and irritability for most of the children.

**CLINICAL TRIAL REGISTRATION NUMBER:**

ACTRN12608000083392.

## RESUMO

**CONTEXTO E OBJETIVO:**

O papel do banho tépido no controle da febre em crianças é controverso. Não encontramos estudos verificando a eficácia do banho tépido associado à dipirona. O objetivo deste estudo foi comparar a eficácia da dipirona associado com banho tépido, com a dipirona isolada no tratamento da febre.

**TIPO DE ESTUDO E LOCAL:**

Foi realizado um ensaio clínico randomizado no hospital de ensino Instituto Materno-Infantil Professor Fernando Figueira, Pernambuco.

**METODOS:**

Foram elegíveis crianças com idade entre 6 a 60 meses, atendidas no setor de emergência com temperatura axilar acima de 38 °C, entre janeiro a julho de 2006. Cento e vinte crianças receberam de forma randomizada, dipirona (20 mg/kg), associada ou não com banho tépido durante 15 minutos. O desfecho primário foi a redução da temperatura axilar, mensurada após 15, 30, 60, 90 e 120 minutos da intervenção; desfechos secundários foram choro e irritabilidade.

**RESULTADOS:**

106 crianças finalizaram o estudo. Nos primeiros 15 minutos, a temperatura diminuiu de forma mais significativa no grupo do banho tépido (p < 0.001). No período de 30 a 120 minutos foi observada maior redução da temperatura no grupo controle. Choro e irritabilidade foram mais observados no grupo estudo, respectivamente, 52% e 36% *versus* nenhuma e duas no grupo controle.

**CONCLUSÕES:**

Banho tépido associado com dipirona baixou de forma mais rápida a temperatura nos primeiros 15 minutos. Ao final dos 120 minutos, observou-se um melhor controle da temperatura com a dipirona isoladamente. Banho tépido provocou moderado desconforto, choro e irritabilidade na maioria das crianças.

## INTRODUCTION

Fever is a common symptom of childhood illness, accounting for 19% to 30% of pediatric emergency visits.^1^ Although fever might be a beneficial physiological response to the infectious process, it can lead to irritability among children and anxiety and parents.^2^ Therefore, physicians usually prefer to treat fever symptomatically. Antipyretic drugs are the main form of treatment, to inhibit the synthesis of prostaglandin, thereby causing less stimulation of the temperature set point in the hypothalamus.^3^ Unlike antipyretics, external cooling acts not by reducing the elevated set point but by overwhelming the metabolically expensive effector mechanisms that have been evoked by the elevated set point.^4^ Physical methods for cooling are often recommended for treating fever and are widely use in some areas. Such treatments include tepid sponging, removing clothing, bathing, fanning and cooling the environment.^5^

Most physical cooling methods are cheap, readily available and frequently used by caregivers, in hospitals and pediatric clinics. However, it is unclear whether physical methods are beneficial, especially when compared with commonly used antipyretic drugs.^6^ There are conflicting results from studies comparing the efficacy and adverse effects of antipyretics and tepid sponging. Some researchers have found that physical methods are less effective than antipyretic drugs for reducing fever and can also cause discomfort, crying and shivering.^7,8^

A Cochrane systematic review found a few small studies demonstrating that tepid sponging alone helps to reduce fever in children.^9^ However, tepid sponging with paracetamol achieves better antipyretic effects than the drug alone.^10-12^ Dipyrone, a pyrazolone nonsteroidal anti-inflammatory agent, is available in many parts of the world, including the Far East, Africa and Latin America. In the latter region, it is the antipyretic that is most used, and its effectiveness and safety have recently been certified in Brazil and Mexico.^13,14^ However because of the risk of agranulocytosis, this drug has been banned in the United States, Canada, Japan and many European countries. In our search in the Medline database (Medical Literature Analysis and Retrieval System Online) and SciELO database (Scientific Electronic Library Online), we did not find any studies using dipyrone and no randomized or quasi-randomized controlled trials comparing it with tepid sponging.

## OBJECTIVE

This study was designed to compare the effects of tepid sponging plus dipyrone with dipyrone alone for reducing fever in children.

## METHODS

A randomized clinical trial to compare tepid sponging plus dipyrone with dipyrone alone for reducing fever in children was undertaken in the Emergency Department of the Instituto Materno Infantil Professor Fernando Figueira (IMIP), Recife, northeastern Brazil, from January to July 2006. IMIP is the most important medical referral centre and the largest children’s hospital in northeastern Brazil.

Children aged six to 60 months who were attended between the hours of 5:00 p.m. and 7:00 p.m., presenting an axillary temperature of between 38.5 °C and 40 °C and a clinical diagnosis consistent with upper respiratory tract infections (URTI), were eligible. Children were included if they were able to stay in the emergency ward for at least two hours. Children requiring admission, urgent investigation or emergency treatment and those who had received antipyretic, steroids or nonsteroidal anti-inflammatory drugs (NSAIDs) during the previous six hours were excluded, as were children with known liver or renal diseases, gastrointestinal bleeding, known allergy to dipyrone, congenital or acquired immunodeficiency or malignancy. The same room was used for all children and informed consent to participate in the trial was obtained from the parents. This study was approved by the local institutional human rights committee (Registration No. 639.05 – CEP/IMIP)

The children were assigned randomly by drawing a numbered envelope to receive oral dipyrone and tepid sponging (sponging group) or dipyrone only (control group). All children were given 20 mg/kg of dipyrone syrup orally at the beginning of the study procedure. The study medication was administered by staff nurses. Children assigned to receive tepid sponging were naked and sponged from head to toe (except the scalp), for 15’. The temperature of the water used ranged from 28 °C to 32 °C. The ambient temperature throughout the period of the study ranged from 27 °C to 30 °C. Axillary temperatures were the primary outcome and were recorded after 15 minutes, 30 minutes, 60 minutes, 90 minutes and 120 minutes. A digital thermometer was used. If, during the course of the study, the child’s body temperature increased by 0.5 °C above baseline the patient was considered to be a treatment failure.

**Figure 1:**
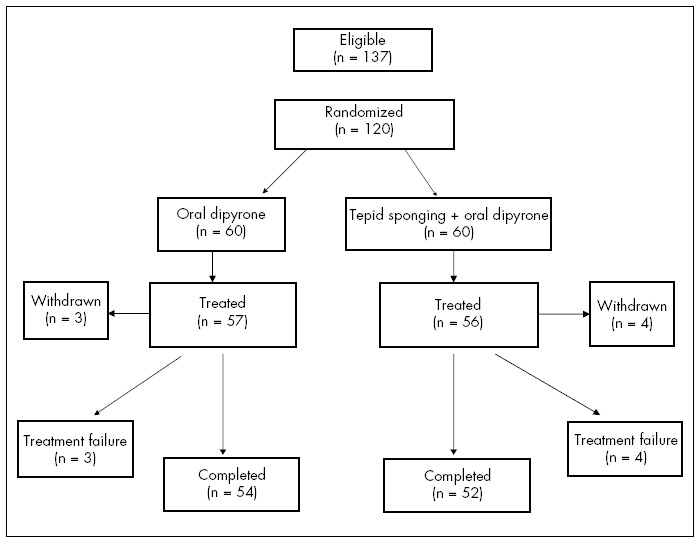
Summary profile for studying the treatment of fever in children using tepid sponging plus dipyrone or dipyrone alone.

It was recorded whether the child was crying, irritable or shivering before each temperature measurement. A summary profile of the study is shown in Figure 1.

The sample size was calculated on the basis of 90% statistical power and an error margin of 0.05. Epi Info version 6 was used to perform the analysis. Student’s t-test was used to compare the means of the axillary temperatures in each group, calculated for each observation time. The chi-squared test was used for categorical variables. A p-value of < 0.05 was used as the cutoff point for statistical significance.

No pharmaceutical laboratories had any role in the study design, data collection or analysis, or writing of the paper.

## RESULTS

During the six-month study period, 876 children were treated for fever in the emergency department between the hours of 5:00 and 7:00 p.m. 756 children were ineligible for the trial: 385 were unable to stay in the emergency ward for a minimum of two hours; 193 fulfilled at least one exclusion criterion, which most often consisted of having received an antipyretic within the previous six hours; and the remaining 178 did not meet the inclusion criteria. Among the 120 eligible children, all were given parental consent for enrollment in the study. Of these, 60 received dipyrone plus tepid sponging and 60 received dipyrone alone (controls). The distribution of the children is summarized in Figure 1. The patients in the two study groups had similar baseline characteristics (Table 1).

**Table 1:** Age, sex, nutritional status, per capita income, fever duration and body temperature of the children at baseline, according to study group (tepid sponging plus dipyrone) and control (dipyrone alone)

Variable	Dipyrone n = 54	TS plus dipyrone n = 52	p
**Age, months (SD)**	21.9 (7.1)	24.9 (8.3)	0.41*
**Sex, n (%)**			
Male	28 (52)	35 (68)	0.15†
Female	26 (48)	17 (32)	
**Nutritional status (%)**			
Low weight/nutritional risk	13 (24)	13 (25)	0.24†
Normal weight	41 (76)	39 (75)	
**Per capita income, (%)**			
< $ 1.00/day	22 (40)	16 (31)	
≥ $ 1.00/day	24 (44)	30 (57)	0.28†
**Unknown**	8 (16)	06 (12)	
**Duration of fever (hours)**	24.5	22.6	0.71*
**Initial temperature °C (SD)**	38.9 °C (0.3)	39.1 °C (0.2)	0.83*

* *Student’s t test;*

† *chi-squared test; TS = tepid sponging, SD = standard deviation.*

The ages of the children in the control group ranged from six to 54 months, and from six to 48 months in the tepid sponging group. There were no statistically significant differences between the study groups with respect to age, sex, nutritional status, temperature, duration of fever on admission and clinical diagnoses. A diagnosis of viral upper respiratory tract infection was made for all these children, based on typical signs (e.g. coryza or pharyngitis) and by ruling out other common causes of fever via clinical assessment.

**Figure 2:**
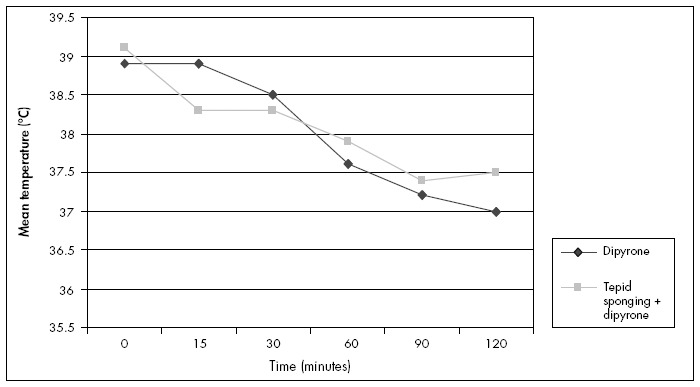
Changes in mean axillary temperature over time, according to use of tepid sponging plus dipyrone or dipyrone alone.

Seven children were withdrawn from the study, all because their parents or guardians were unable to continue waiting in the emergency ward for two hours, of whom four were in the study group and three were in the control group. There were seven cases of treatment failure: four in the study group and three in the control group. Fifteen minutes after the baseline time, the axillary temperature fall was significantly greater in the sponged group than in the control group (p < 0.001). Over the period from 30 to 120 minutes after the baseline time, the axillary temperature fell in both groups between each observation point, and there were significant differences between the groups at the 60-minute and 120-minute axillary temperature measurements (Table 2 and Figure 2). The mean temperature reduction from time zero to 120 minutes was significantly greater in the sponged group only at 15 minutes. From 30 minutes to 120 minutes, the mean temperature reduction was greater in the control group (Table 3). The mean temperature decreased gradually from 39.1 ºC to 37.5 ºC in the sponged group and from 39.1 ºC to 37.0 ºC in the control group.

**Table 2:** Axillary temperature at each time point, for sponged group and control group

Time minutes	Group	Temperature						p*
		n	Mean	SD	Minimum	Median	Maximum	
Baseline	Dipyrone	54	38.9	0.4	38.5	38.8	39.9	0.080
	TS plus dipyrone	52	39.1	0.3	38.5	39.1	39.5	
15	Dipyrone	54	38.9	0.5	38.0	38.9	40.0	< 0.001
	TS plus dipyrone	52	38.3	0.5	37.4	38.3	39.1	
30	Dipyrone	54	38.5	0.5	37.6	38.5	39.6	0.117
	TS plus dipyrone	52	38.3	0.6	37.0	38.3	39.2	
60	Dipyrone	54	37.6	0.4	36.6	37.7	38.3	0.002
	TS plus dipyrone	52	37.9	0.5	36.8	38.0	39.1	
90	Dipyrone	54	37.2	0.7	35.7	37.1	38.3	0.425
	TS plus dipyrone	52	37.4	0.5	36.0	37.3	38.3	
120	Dipyrone	54	37.0	0.6	36.0	37.1	37.8	0.022
	TS plus dipyrone	52	37.5	0.5	36.6	37.6	38.2	

* *Student’s t test; TS = tepid sponging; SD = standard deviation.*

**Table 3:** Mean temperature variation (in °C ± SD) between measurement times from 0 to 120 minutes

Times (minutes)	Dipyrone (n = 54)	Tepid sponging plus dipyrone (n = 52)	95% CI	p
0 – 15	- 0.01 ± 0.38	- 0.78 ± 0.46	0.53 – 1.01	0.001
15 – 30	- 0.38 ± 0.49	- 0.02 ± 0.65	0.03 – 0.69	0.034
30 – 60	- 0.86 ± 0.41	- 0.39 ± 0.38	0.24 – 0.70	0.001
60 – 90	- 0.41 ± 0.48	- 0.56 ± 0.37	0.44 – 0.14	0.307
90 – 120	- 0.13 ± 0.271	- 0.02 ± 0.23	0.38 – 0.64	0.601

*SD = standard deviation; CI = confidence interval.*

Crying was observed in 52% of the sponged children and in none of the controls. Irritability was observed in 36% of the sponged children and in only two of the controls. Shivering was observed in only one child, a one-year-old who had had fever for 12 hours.

## DISCUSSION

Tepid sponging, in addition to oral dipyrone, was more effective in reducing temperature during the first 15 minutes after drug administration than was dipyrone alone. We did not find any other study comparing tepid sponging plus dipyrone with dipyrone alone. The controversial association of dipyrone with agranulocytosis has led to the banning or withdrawal of this drug from the market in most developed countries.^15^ This has limited the number of studies on the safety and efficacy of this antipyretic. However, studies with paracetamol have reported that the reduction in fever was greater when sponging was combined with oral paracetamol than when paracetamol was used alone.^16-18^

Our results were similar to those from randomized controlled trials on tepid sponging compared with oral paracetamol (15 mg/kg) that found sponging more effective in reducing body temperature only during the first 30 minutes of treatment.^8,9,11^ In a review, Bernath et al. concluded that tepid sponging appears to be more effective within the first 30 minutes of treatment and has an additive effect when combined with paracetamol.^18^ In some studies, the effects of paracetamol or ibuprofen became superior to sponging after 60 minutes.^8,11^ In our study, we also observed lower axillary temperatures with dipyrone alone after this length of time, and there was also an increase in axillary temperature after 120 minutes. This can be explained as a rebound reaction because, unlike antipyretics, external cooling acts not by reducing the elevated set point but by overwhelming the metabolically expensive effector mechanisms that have been evoked by the elevated set-point.^19^

Tepid sponging may have more benefit in tropical climates because heat is not readily lost to the environment following antipyretic medication.^12^ Our findings in febrile children in a tropical climate showed that the reduction in temperature was much greater when, in addition to dipyrone, heat loss from the body was promoted by tepid sponging. Even without antipyretics, tepid sponging is often used to reduce fever. Some studies have suggested that this is effective only during the first 30 minutes and that paracetamol is clearly more effective than tepid sponging in reducing body temperature in febrile children in a tropical climate.^16,17^ We believe that tepid sponging could be specially addressed to children with a risk of febrile seizures. For patients with hyperthermia, external cooling may be lifesaving.^20^

We found mild discomfort (crying) caused by tepid sponging. Mahar et al.^12^ showed that crying was associated with sponging combined with antipyretic medication and that fewer children cried if sponging was performed by their parents. According to Axelrod,^8^ febrile children treated with tepid-water sponging plus antipyretic drugs are more uncomfortable that those treated with antipyretic drugs alone, although they exhibit slightly more rapid reductions in temperature.

## CONCLUSION

In conclusion, when a rapid temperature reduction is required in a febrile child in a tropical environment, tepid sponging in addition to dipyrone provided faster cooling during the first 15 minutes, but dipyrone alone gave better temperature control over the two-hour period.
